# Phase II study of sequential S-1 and cyclophosphamide therapy in patients with metastatic breast cancer

**DOI:** 10.1186/s12885-020-07550-5

**Published:** 2020-11-06

**Authors:** Keiko Yanai, Takaaki Fujii, Jun Horiguchi, Yuko Nakazawa, Sasagu Kurozumi, Sayaka Obayashi, Reina Yajima, Ken Shirabe

**Affiliations:** 1 Division of Breast and Endocrine Surgery, Department of General Surgical Science, Graduate School of Medicine, Gunma University, Gunma, Japan; 2 Department of General Surgical Science, Graduate School of Medicine, Gunma University, Gunma, Japan; 3 Breast Surgery, International University of Health and Welfare, Chiba, Japan

**Keywords:** Metastatic breast cancer, S-1, Cyclophosphamide

## Abstract

**Background:**

S-1 and cyclophosphamide (CPA) can be given orally, and their combination may have great potential for treating metastatic breast cancer (MBC). A phase I study of sequential S-1 and CPA therapy was conducted in patients with MBC; the recommended doses that were determined for this regimen were 80 mg/m^2^/day for S-1 and 100 mg/m^2^/day for CPA. We then conducted a phase II study of this oral S-1 and CPA regimen.

**Methods:**

This was a single-arm, open-label, single-center prospective phase II study to evaluate the efficacy of a sequential S-1 and CPA regimen for MBC. S-1 was administered orally 2×/day for 14 consecutive days, and then CPA was administered orally 2×/day for 14 consecutive days in a repeating 4-week cycle (S-1 for 2 weeks, CPA for 2 weeks). The primary endpoint was the overall response rate (ORR). Secondary endpoints included the overall survival (OS), progression-free survival (PFS), clinical benefit rate (CBR) and safety.

**Results:**

Thirty-six patients were enrolled in this study. The overall response was complete response in 0 (0%), partial response in 12 (33.3%), stable disease in 12 (33.3%), and progressive disease in 11 (30.1%) patients. The ORR was 33.3% (12/36). The CBR was 66.7% (24/36). The median PFS was 9.5 months (95%CI: 7.8–12.6 months). The median OS was 20.2 months (95%CI: 15.0–25.4 months) Grade 3/4 adverse events included leukopenia in seven patients (19.4%). Dose reductions because of adverse events occurred in 12 patients (33.3%). There was no treatment-related mortality.

**Conclusion:**

The combination of sequential therapy with S-1 and CPA was tolerable and had efficacy with good disease control. Sequential therapy with S-1 and CPA may be a feasible new treatment option for patients with MBC; however, further study is warranted to explore the efficacy of this therapy.

**Trial registration:**

JRCT, JRCTs031180296. Registered 2 December 2019 – Retrospectively registered.

## Background

Metastatic breast cancer (MBC) is not yet curable. The current treatment strategies for patients with MBC simply prolong survival and improve or maintain the patient’s quality of life (QOL) [1–4]. There is an increasing demand for effective regimens that have less adverse effects for MBC patients. Taxane- or anthracycline-based regimens are established cytotoxic agents for patients with MBC, but the administration of taxane or anthracycline can cause serious adverse events (including myelosuppression, hair loss, nausea, edema, and peripheral neuropathy) that may affect patients’ health-related quality of life (HRQOL) [4, 5]. Less-toxic treatments that do not reduce the HRQOL are needed for the management of MBC.

Orally administered drugs are generally more convenient to use than intravenous drugs [4, 6]. The oral fluorouracil derivatives S-1 is widely used in Japan [3, 4, 7–10]. S-1 is a combination of tegafur (a prodrug of 5-fluorouracil), gimeracil (an inhibitor of dihydropyrimidine dehydrogenase, the rate-limiting enzyme in the catabolism of 5-fluorouracil), and potassium oxonate (an inhibitor of orotate phosphoribosyltransferase, which suppresses the gastrointestinal toxicity of 5-fluorouracil) in the molar ratio of 1.0: 0.4:  1.0 [2, 3]. S-1 treatment resulted in an overall response rate of 41.7% in a phase II trials of patient with breast cancer in Japan [8].

A recent randomized phase III study (the SELECT BC trial) indicated that as a first-line treatment for MBC, S-1 is noninferior to taxane with respect to overall survival [4]. We also reported that the combination of S-1 and trastuzumab was tolerable and had excellent efficacy with good response and disease control for HER2-positive metastatic breast cancer [3]. Regarding adverse events, S-1 has shown low incidences of myelosuppression, nausea, vomiting, alopecia, and peripheral neuropathy [2, 3]. Thus, S-1 was demonstrated to have high efficacy for MBC with a low incidence of adverse events [4, 8].

Cyclophosphamide (CPA) is one of the oldest drugs used in oncology; it was the first available drug in oral formulations for the management of MBC [11–13]. CPA is typically used as a component of combination regimens such as AC (doxorubicin and CPA) and fluoropyrimidine-based combination regimens including CMF (CPA, methotrexate, and 5-fluorouracil [5-FU]) [14]. The use of oral agents can make a significant contribution to a patient’s QOL. Several studies reported that oral combination regimens of CPA plus UFT (tegafur/uracil) or capecitabine is effective with well-tolerated toxicities in patients with MBC [15–17]. However, no data evaluating the efficacy of S-1 plus CPA therapy for MBC is available in the existing literature. We thus conducted a phase I study of sequential S-1 and CPA therapy to determine the dose-limiting toxicities (DLTs) and recommended doses (RDs) in patients with MBC [18], and we reported that sequential therapy with S-1 and CPA could be safely and effectively used for the treatment of MBC; the RDs determined for this regimen were 80 mg/m^2^/day for S-1 and 100 mg/m^2^/day for CPA [18]. We then performed a phase II trial to verify the clinical efficacy of sequential S-1 and CPA for MBC, and we report the results as follows.

## Methods

### Study design and patients

We conducted a single arm, open-label, single center phase II trial to evaluate the efficacy of a sequential S-1 and CPA regimen for MBC. Women aged 20–75 years old with a histological diagnosis of MBC were considered eligible for the study. Eligibility required a measurable tumor based on the RECIST criteria; an Eastern Cooperative Oncology Group performance status of 0 or 1; a body surface area > 1.25; expected survival > 3 months; adequate organ function defined as a leukocyte count 3500–12,000/mm^3^ (or neutrophil count > 2000/mm^3^), platelet count > 100,000/mm^3^, hemoglobin > 9 g/dl, serum ALT and AST level less than the upper level of normal in each institution × 2.5, serum total bilirubin < 1.5 mg/dl, and serum creatinine < 1.2 mg/dl (or creatinine clearance > 50 ml/min); and the resolution of all toxicities from prior therapy. All patients provided written informed consent to participate in this study. The sample size was estimated to be approximately 40 patients, without any calculations based on statistical assumptions from previous phase I/II studies [10–15].

The exclusion criteria included symptomatic central nervous system metastases, active systemic infectious disease, clinically significant cardiovascular impairment, serious concomitant illness, pregnancy, breast feeding, a history of other cancers with a disease-free interval of ≤5 years, patients who had received trastuzumab-containing therapy within the prior 4 weeks, those who were receiving 5-FU-containing therapy, flucytosine, or pentostatin, and those who had recurrent disease within 1 year after having received 5-FU-containing therapy or CPA. From November 2007 to December 2018, a total of 36 patients were enrolled in this study. The demographic characteristics of the patients are summarized in Table 1. The study was carried out at Gunma University, Japan. The study protocol was approved by our Ethics Committee.

**Table 1:** Patients’ characteristics and clinicopathological features

Age median (range), (y.o.)	50 (33–74)
Metastatic de-novo or recurrent, (n)	
Metastatic de-novo	10 (27.8%)
Metastatic recurrent	26 (72.2%)
Subtype	
Luminal	29 (80.6%)
Triple negative	7 (19.4%)
Number of previous chemotherapy regimens, (n)	
0	22 (61.1%)
1	10 (27.8%)
2	2 (5.6%)
≧3	2 (5.6%)
Previous endocrine therapy, (n)	26 (72.2%)
Metastatic sites, (n)	
Visceral	23 (63.9%)
Non-visceral	13 (36.1%)

### Treatment procedure

According to the RDs determined by our phase I study [18], S-1 80 mg/m^2^/day divided twice a day orally for 14 consecutive days, and then CPA 100 mg/m^2^/day divided twice a day orally for 14 consecutive days in a repeating 4-week cycle (S-1 for 2 weeks, CPA for 2 weeks). This regimen was continued until the occurrence of (1) progressive disease (PD) as assessed by the investigator using RECIST criteria, or (2) the appearance of unmanageable toxicity, or (3) the patient’s withdrawal of consent. Any concomitant medication could be given at the discretion of the investigator if it was considered necessary for the patient’s welfare and was not expected to interfere with the evaluation of the study treatment. Other antitumor therapies were not permitted.

### Assessments

The primary endpoint was the antitumor activity of the sequential S-1 and CPA therapy as assessed by the overall response rate (ORR) based on the RECIST criteria. The secondary endpoints included the patients’ overall survival (OS) and progression-free survival (PFS), the clinical benefit rate (CBR), and the regimen’s safety. Routine tumor assessments based on the RECIST criteria were performed 1 month after the first dose and then every month during the treatment period. The ORR was defined as the proportion of patients with a complete response (CR) or a partial response (PR) among all patients. The CBR was defined as the proportion of patients with a CR or PR or stable disease (SD) continuing > 4 weeks (28 days). CR and PR required confirmation at ≥4 weeks after first being reported. The Kaplan-Meier approach was used to estimate the median PFS and OS values.

Treatment-related adverse events were evaluated according to the Common Terminology Criteria for Adverse Events, ver. 3.0 and 4.0. There was a protocol amendment that changed the evaluation from version 3 to 4 in the middle of the trial. The incidence of adverse events was calculated according to grade. Hematology and biochemistry assessments, physical examinations, and periodic measurements of vital signs were performed before the start of each treatment cycle.

## Results

### The patient’s characteristics

From November 2007 to December 2018, a total of 36 patients were enrolled in this study at Gunma University, Japan. The characteristics of patients are summarized in Table 1. Of the 36 patients, 26 had metastatic recurrent cancer, and 10 patients had metastatic de-novo breast cancer. The median age was 50 years (range 33–74). Twenty-nine patients were hormonal receptor (HR)-positive (ER+ and/or PgR+). Seven patients had triple-negative tumors. Visceral metastasis including liver and lung metastases was observed in 23 cases (63.9%), and non-visceral metastasis including bone and lymph nodes metastases was observed in 13 cases (36.1%). Ten patients had received one line of chemotherapy before registration; 2 other patients had received 2 lines of chemotherapy, and another 2 patients had received ≥3 of chemotherapy. Thus, 22 patients (61.1%) were receiving sequential S-1 and CPA treatment as their first-line chemotherapy. Twenty-six patients received endocrine therapy for MBC before registration in this study.

### Tumor responses and survival

**Fig. 1:**
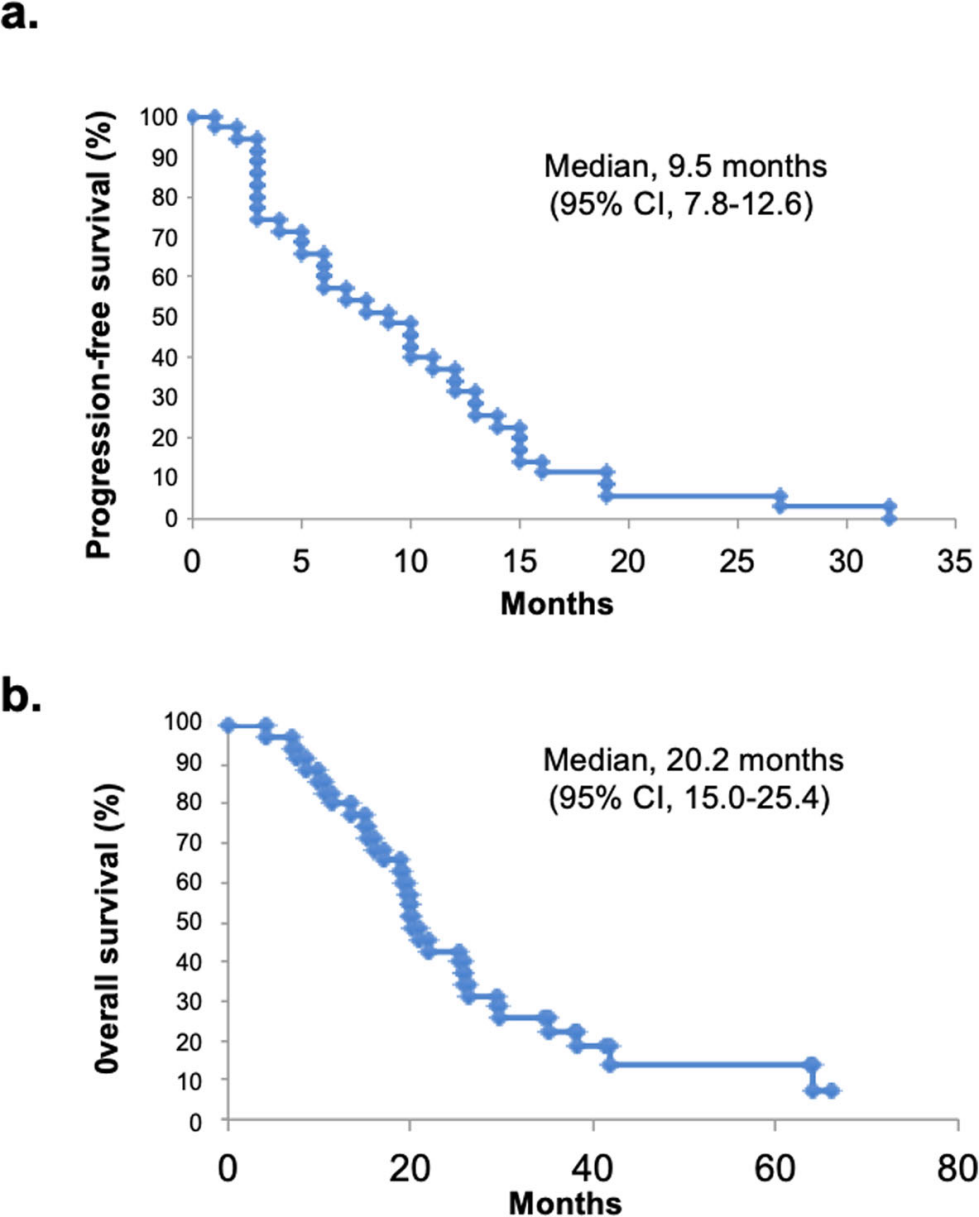
Kaplan-Meier estimates of the progression-free survival (PFS) and overall survival (OS). **a** The median PFS was 9.5 months (95% CI: 7.8–12.6 months). **b** The median OS was 20.2 months (95% CI: 15.0–25.4 months)

The overall response was a CR in 0 (0%), a PR in 12 (33.3%), SD in 12 (33.3%), and PD in 11 (33.3%) patients; one patient was non-evaluable for response. The ORR was 33.3% (12/36), and the CBR was 66.7% (24/36). The median PFS in this patient population was 9.5 months (95%CI: 7.8–12.6 months), and the median OS was 20.2 months (95%CI: 15.0–25.4 months) (Fig. 1).

**Fig. 2:**
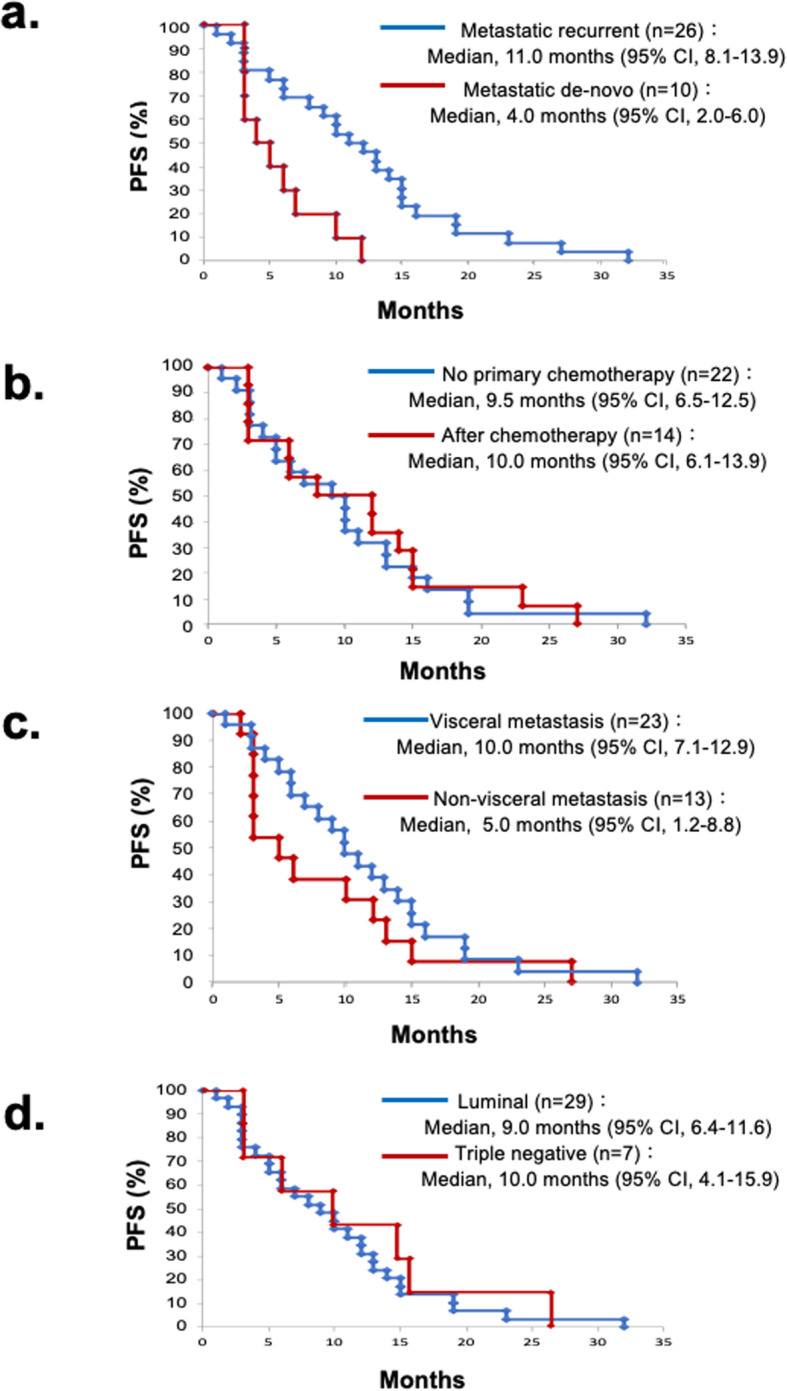
Kaplan-Meier estimates of the progression-free survival (PFS) of subgroup analysis. **a** Metastatic de-novo versus metastatic recurrent. The median PFS was 11.0 months (95%CI: 8.1–13.9 months) in patients with metastatic recurrent, and the median PFS was 4.0 months (95%CI: 2.0–6.0 months) in patients with metastatic de-novo. **b** No prior chemotherapy versus after chemotherapy. The median PFS was 9.5 months (95%CI: 6.5–12.5 months) in patients with no primary chemotherapy, and the median PFS was 10.5 months (95%CI: 6.1–13.9 months) in patients after chemotherapy. **c** Visceral metastasis versus non-visceral metastasis. The median PFS was 10.0 months (95%CI: 7.1–12.9 months) in patients with visceral metastasis, and the median PFS was 5.0 months (95%CI: 1.2–8.8 months) in patients without visceral metastasis. **d** Luminal type versus triple negative breast cancer. The median PFS was 9.0 months (95%CI: 6.4–11.6 months) in patients with luminal type, and the median PFS was 10.0 months (95%CI: 4.1–15.9 months) in patients without triple negative breast cancer. There were no significant differences between patients with and without prior chemotherapy (*p* = 0.784), visceral metastasis (*p* = 0.254) or subtype (*p* = 0.609), however, the PFS was significantly shorter in patients with metastatic de-novo than that in patients with metastatic recurrent (*p* = 0.007)

We divided the patients into two groups based on a) metastatic de-novo versus metastatic recurrent; b) with no prior chemotherapy versus those with one or more of them; c) with visceral metastasis versus non-visceral metastasis; d) luminal type versus triple negative breast cancer (Table 2a-d). There were no significant differences between patients with and without prior chemotherapy (*p* = 0.784), visceral metastasis (*p* = 0.254) or subtype (*p* = 0.609); however, the PFS was significantly shorter in patients with metastatic de-novo disease than that in patients with metastatic recurrent disease (*p* = 0.007) (Fig. 2a-d).

**Table 2:** Patients’ characteristics and clinicopathological feature in subgroup analysis.

a) Metastatic de-novo versus metastatic recurrent			
	Metastatic recurrent (*n* = 26)	Metastatic de-novo (*n* = 10)	*P* value
Age median (range), (y.o.)	51 (33-74)	49 (35-64)	0.344
Subtype			0.079
Luminal	19 (73.1%)	10 (100%)	
Triple negative	7 (26.9%)	0 (0%)	
Number of previous chemotherapy regimens, (n)			0.515
0	17 (65.4%)	5 (50.0%)	
1	6 (27.3%)	4 (40.0%)	
2	1 (3.8%)	1 (10.0%)	
≧3	2 (7.7%)	0 (0%)	
Previous endocrine therapy, (n)	16 (61.5%)	10 (100%)	0.021
Metastatic sites, (n)			0.473
Visceral	16 (61.5%)	7 (70.0%)	
Non-visceral	10 (27.8%)	3 (30.0%)	
b) No prior chemotherapy (CT) versus after chemotherapy.			
	No primary CT (*n* = 22)	After CT (*n* = 14)	*P* value
Age median (range), (y.o.)	51 (34-74)	47 (33-73)	0.243
Metastatic de-novo or recurrent, (n)			0.318
Metastatic de-novo	5 (22.7%)	5 (35.7%)	
Metastatic recurrent	17 (77.3%)	9 (64.3%)	
Subtype			0.433
Luminal	17 (77.3%)	12 (85.7%)	
Triple negative	5 (22.7%)	2 (14.3%)	
Previous endocrine therapy, (n)	16 (72.7%)	10 (71.4%)	0.611
Metastatic sites, (n)			0.374
Visceral	15 (68.2%)	8 (57.1%)	
Non-visceral	7 (31.8%)	6 (42.9%)	
c) Visceral metastasis versus non-visceral metastasis.			
	Visceral metastasis (*n* = 23)	Non-visceral metastasis (*n* = 13)	*P* value
Age median (range), (y.o.)	51.5 (33-73)	48 (34-74)	0.135
Metastatic de-novo or recurrent, (n)			0.473
Metastatic de-novo	7 (30.4%)	3 (23.1%)	
Metastatic recurrent	16 (69.6%)	10 (76.9%)	
Subtype			0.499
Luminal	19 (82.6%)	10 (76.9%)	
Triple negative	4 (17.4%)	3 (23.1%)	
Number of previous chemotherapy regimens, (n)			0.920
0	14 (60.9%)	8 (61.5%)	
1	7 (30.4%)	3 (23.1%)	
2	1 (4.3%)	1 (7.7%)	
≧3	1 (4.3%)	1 (7.7%)	
Previous endocrine therapy, (n)	18 (78.3%)	8 (61.5%)	0.420
d) Luminal type versus triple negative breast cancer.			
	Luminal (*n* = 29)	TN (*n* = 7)	*P* value
Age median (range), (y.o.)	50 (33-74)	58 (36-73)	0.219
Metastatic de-novo or recurrent, (n)			0.079
Metastatic de-novo	10 (22.7%)	0 (35.7%)	
Metastatic recurrent	19 (77.3%)	7 (64.3%)	
Number of previous chemotherapy regimens, (n)			0.773
0	17 (60.9%)	5 (61.5%)	
1	8 (30.4%)	2 (23.1%)	
2	2 (4.3%)	0 (7.7%)	
≧3	2 (4.3%)	0 (7.7%)	
Metastatic sites, (n)			0.499
Visceral	19 (68.2%)	4 (57.1%)	
Non-visceral	10 (31.8%)	3 (42.9%)	
	Metastatic recurrent (*n* = 19)	Metastatic de-novo (*n* = 10)	*P* value
Age median (range), (y.o.)	50 (33-74)	49 (35-64)	0.618
Number of previous chemotherapy regimens, (n)			0.510
0	12 (65.4%)	5 (50.0%)	
1	4 (27.3%)	4 (40.0%)	
2	1 (3.8%)	1 (10.0%)	
≧3	2 (7.7%)	0 (0%)	
Previous endocrine therapy, (n)	15 (61.5%)	10 (100%)	0.163
Metastatic sites, (n)			0.522
Visceral	12 (61.5%)	7 (70.0%)	
Non-visceral	7 (27.8%)	3 (30.0%)

### Safety

All patients were assessed for toxicities during the treatment cycles. The adverse events are shown in Table 3. Regarding hematologic toxicity, leukopenia occurred in seven (19.4%) patients, anemia in one (2.8%), and thrombocytopenia in three (8.3%). Five (13.9%) patients had grade 3 leukopenia, but no patients had grade 4 hematologic toxicity. Grade 3/4 adverse events included leukopenia in 7 patients each (19.4%). With regard to non-hematological toxicities, the most common adverse event was fatigue. One patient had nasolacrimal duct obstruction. Dose reductions because of adverse events occurred in 12 patients (33.3%). Treatment was discontinued because of sepsis in one patient (2.8%). There was no treatment-related mortality.

**Table 3:** The treatment-related any-grade adverse events and grade 3/4 adverse events

Adverse Events, (n)	All Grade	Grade 3/4
Leukopenia	7 (19.4%)	5 (13.9%)
Anemia	1 (2.8%)	1 (2.8%)
Thrombocytopenia	3 (8.3%)	1 (2.8%)
Fatigue	3 (8.3%)	0
Nasolacrimal duct obstruction	1 (2.8%)	0
Sepsis	1 (2.8%)	1 (2.8%)

## Discussion

The goals of the current treatment for patients with MBC are to prolong survival and improve or maintain an adequate QOL and HRQOL [1–4]. Thus, less-toxic treatments should be chosen as long as the treatment can control the disease progression [9]. Compared to intravenous chemotherapy for patients with MBC, the use of oral chemotherapy affords a better QOL [4, 6]. S-1 chemotherapy, which is often used in Japan, is composed of oral fluorouracil derivatives. In the SELECT BC trials, S-1 was shown to be noninferior to taxane with respect to OS and better than taxane with regard to HRQOL as a first-line treatment for patients with MBC [4]. In addition, 5-FU demonstrated a synergistic antitumor effect in combination with CPA in experimental studies and in a phase II trial [14, 19–22], but it also had a significantly higher rate of toxicity [21, 22].

The present study’s ORR and CBR were 33.3 and 66.7%, respectively, with 9.5 months as the median PFS of and 20.2 months as median OS. The treatment was well tolerated. The most common toxicity was leukopenia, which was observed in 19.4% of cases. Previous phase II studies using standard metronomic chemotherapy revealed that leukopenia was observed in 51% or 31% [12, 13] and thrombocytopenia was observed in 5 and 8% [12, 13], which is considered to be same to the toxicity of this study. These results strongly suggest that sequential S-1 and CPA therapy is an effective treatment option for MBC, with a manageable toxicity profile. In the SELECT BC trial, the median time to failure (TTF) was 8.0 months in the group administered S-1 as the first-line chemotherapy [4]. Regarding capecitabine+CPA combination treatment, two phase II studies have already reported this regimen’s efficacy [15, 16]. The ORRs of 35.6 and 30.3% in those studies are consistent with our present study. The median PFS in those studies were 6.6 months and 5.2 months, respectively [15, 16].

There is no meaningful biomarker for the efficacy of S-1/CPA. We conducted the comparison of the data obtained in certain subgroups of patients in order to find out patients with better chances of having a good clinical response. We divided the patients into two groups based on a) metastatic de-novo versus metastatic recurrent; b) with no prior chemotherapy versus those with one or more of them; c) with visceral metastasis versus non-visceral metastasis; d) luminal type versus triple negative breast cancer. There were no significant differences between patients with and without prior chemotherapy, visceral metastasis or subtype, however, the PFS was significantly shorter in patients with metastatic de-novo than that in patients with metastatic recurrent, suggesting that this therapy may show superior effect in patients with metastatic recurrent. The mechanism is unknown, however, all patients with metastatic de-novo were luminal type. Further study should be done to evaluate how metastatic de-novo breast cancer influence the anti-tumor effect of S-1/CPA in large number of patients.

All-grade leukopenia was observed in 19.4% of our present series, and one patient was unable to continue the therapy. In the SELECT BC trial, all-grade leukopenia was observed in 43% of the S-1 group [4]. Of our 36 patients with MBC, grade 3 leukopenia was observed in 13.9%, as one of the most common adverse events in this regimen. Adverse events such as hair loss, peripheral neuropathy, gastrointestinal toxicity and edema — which are commonly observed in patients with taxane or anthracycline regimens [23] — were not observed in our study. The benefit of avoiding hair loss is of particular concern from the patients’ perspective [24]. Approximately 10–20% of patients who received S-1 developed lacrimal drainage obstruction or stenosis [25, 26]. In the present trial, only one (2.8%) patient developed lacrimal drainage obstruction. In light of these results, we suggest that this sequential S-1 and CPA therapy is a feasible and tolerable regimen in terms of both efficacy and safety.

This study has potential limitations, the major one being the small number of cases (*n* = 36) and the inclusion of a single center. However, this is the first prospective clinical trial to evaluate the efficacy of sequential therapy with S-1 and CPA for metastatic breast cancer. Additional research is needed to explore the efficacy of this therapy in larger numbers of patients to confirm the effects and safety profile of sequential therapy with S-1 and CPA.

## Conclusions

The combination of sequential therapy with S-1 and CPA was tolerable and had efficacy with good disease control in this study. Sequential therapy with S-1 and CPA may be a feasible new treatment option for patients with MBC. Further study is warranted to explore the efficacy of sequential therapy with S-1 and CPA, especially considering this is a small, open-label, single center trial with no formal hypothesis testing.

## Data Availability

The datasets used and/or analysed during the current study are available from the corresponding author on reasonable request.

## Abbreviations

CBR: Clinical benefit rate
CPA: Cyclophosphamide
CR: Complete response
DLTs: Dose-limiting toxicities
HRQOL: Health-related quality of life
MBC: Metastatic breast cancer
ORR: Overall response rate
OS: Overall survival
PD: Progressive disease
PFS: Progression-free survival
PR: Partial response
QOL: Quality of life
RDs: Recommended doses
SD: Stable disease
TTF: Time to failure
